# Extramedullary Acute Leukemia—Still an Unforeseen Presentation

**DOI:** 10.3390/hematolrep14020021

**Published:** 2022-04-18

**Authors:** Dina Rochate, Carolina Pavão, Rui Amaral, Carolina Viveiros, José Cabeçadas, Vitor Carneiro, Cristina Fraga

**Affiliations:** 1 Divino Espírito Santo’s Hospital, 9500-370 Ponta Delgada, Portugal; carolina.mcpavao@gmail.com (C.P.); amaral_rui@hotmail.com (R.A.); cviveiros82@gmail.com (C.V.); gvcarneiro@gmail.com (V.C.); cristinafragabarros@gmail.com (C.F.); 2 Portuguese Institute of Oncology, 1070-212 Lisbon, Portugal; jcabecadas@ipolisboa.min-saude.pt

**Keywords:** acute myeloid leukemia, extramedullary tumor, myeloid sarcoma, leukemia cutis

## Abstract

Myeloid sarcomas (MS) are rare extramedullary (EM) hematological tumors that generally arise during the natural course of acute myeloid leukemia (AML), occurring concomitantly with the onset of systemic leukemia; it can also occur following onset but rarely before. Common sites of EM involvement include the lymph nodes, skin, soft tissue, bone and peritoneum. Herein, we report the case of a 63-year-old man who presented EM AML upon initial diagnosis involving the bone marrow, lymph nodes and skin (leukemia cutis). A diagnosis was made based on immunohistochemistry (IHC). This case presents a diagnostic dilemma due to its atypical presentation and the sites involved. It also highlights the importance of IHC in the diagnosis of EM AML. The potential role of hypomethylating agents and Venetoclax in cases not eligible for hematopoietic stem cell transplant are also discussed.

## 1. Introduction

Hematological malignancies may manifest as extramedullary soft tissue masses that can occur at sites other than the bone marrow. Extramedullary (EM) manifestations of acute myelogenous leukemia (AML) are described in the literature as occurring in 2–9% of newly diagnosed AML [1,2]. Myeloid sarcoma (MS) (also known as chloroma or granulocytic sarcoma) is an EM tumor mass composed of malignant primitive myeloid cells that infiltrate and efface the underlying tissue structure [3]. In the event of skin manifestations, EM AML is usually referred to as leukemia cutis (LC). LC is an infiltration of the epidermis, dermis or subcutis by leukemia cells and commonly presents as a papulonodular rash in 3% of patients with AML [4,5]. In the 2016 revision of the World Health Organization’s classification of myeloid neoplasms and acute leukemia, MS is described as occurring in isolation or, more commonly, in those with a history of AML [3,6]. MS has a slight male predominance and may occur at any site, leading to varied clinical presentations, but most commonly, it affects the lymph nodes, skin, soft tissues, bone and peritoneum [3,5]. Due to its rarity, MS is a diagnostic and therapeutic challenge. In retrospective studies, the misdiagnosis rate is 25–47%, with cases being commonly misidentified as malignant lymphoproliferative disorders [7]. The combination of radiology, histology, immunophenotyping and molecular analyses is essential for diagnosis and treatment planning. We present a case report of EM AML, which upon initial diagnosis, involved the bone marrow, lymph nodes and skin. Diagnostic challenges and therapeutic issues are discussed.

## 2. Case Report

A 63-year-old man was admitted to the emergency department after having suffered from night sweats and weight loss for 3 weeks. His previous medical history included lumbar disc pathology and unilateral leg pain; he was also a heavy smoker (55 pack-years). Upon admission, the patient was hemodynamically stable and afebrile, presenting ECOG 1. A general examination revealed pallor, bilateral enlarged lymph nodes (cervical, axillary and inguinal) and palpable hepatomegaly. The cutaneous examination showed purple/reddish patches on the upper body. Laboratory tests showed pancytopenia with severe neutropenia and increased lactate dehydrogenase (Table 1).

**Figure 1:**
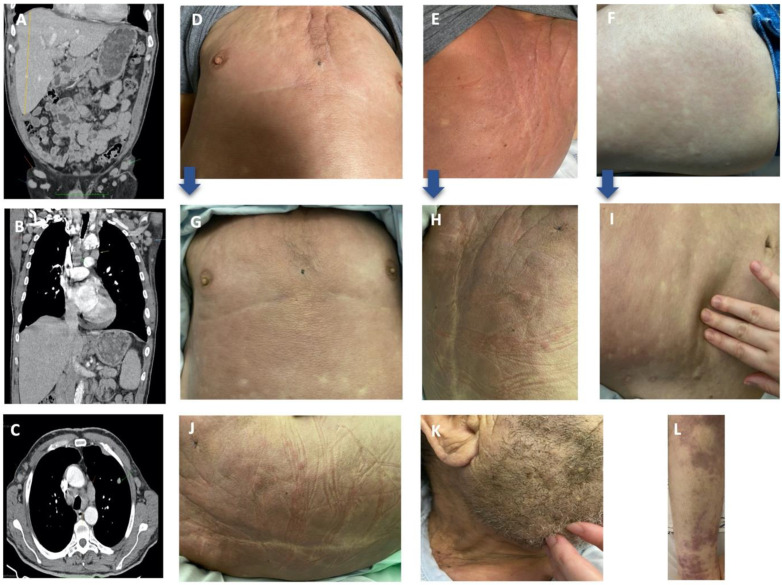
Imaging (**A**–**C**) and clinical (**D**–**I**) findings: (**A**–**C**) CT scan (axial and coronal reformations) showing hepatomegaly and lymphadenopathy (inguinal, axillary and mediastinal) with no splenomegaly. Noteworthy are the focal pleural thickening and pulmonary nodule (left upper lobe with 12 mm); (**D**–**F**) cutaneous examination shows diffuse erythema and skin thickening in upper trunk with scattered nodules; and (**G**–**I**) marked cutaneous infiltration with progressive aggravation of the skin thickening on the upper trunk. Blue arrows show the evolution in the images from top to bottom; clinical aggravation with marked cutaneous infiltration with aggravated skin thickening in upper and lower trunk (**J**), neck (**K**) and new purple patches on lower limb (**L**).

A peripheral blood smear revealed the apparent presence of blasts. The patient was admitted with suspected hematological malignancy. Empirical antibiotic therapy with amoxicillin and clavulanic acid was administered along with folic acid. Viral serology and blood cultures were negative. A computed tomography scan (CT) confirmed hepatomegaly and multiple mediastinal, axillary, celiac, hepatic, inguinal and mesenteric lymphadenopathy with hepatomegaly but no splenomegaly (Figure 1A,B). A thoracic CT scan showed focal pleural thickening in the anterior aspect of the left hemithorax and nodular formation (12 mm) probably as an outcome of underlying disease (Figure 1C).

**Figure 2:**
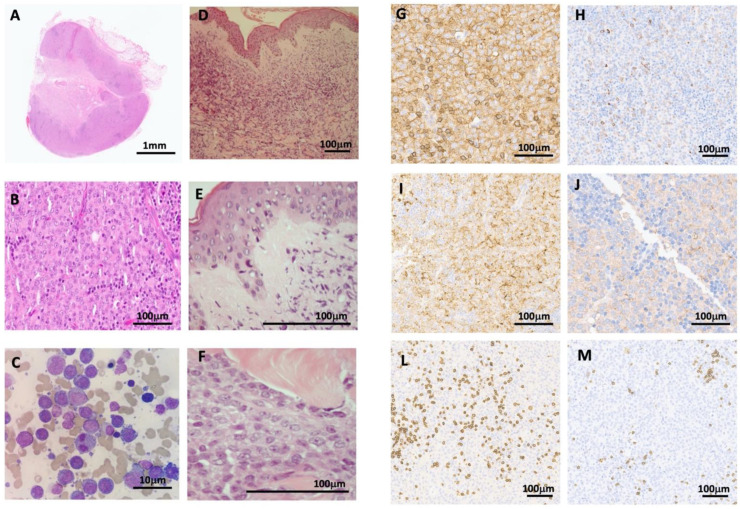
Laboratory findings. (**A**,**B**) Lymph node biopsy: structural changes with diffuse proliferation. The cells are large with large nuclei and evident nucleoli. The cytoplasm is vast and eosinophilic. (**A**) HE ×10; (**B**) HE ×400. (**C**) Bone marrow massively infiltrated by blast cells. May–Grunwald ×100. (**D**,**E**) Skin biopsy: there is a monotonous interstitial cellular infiltrate that spares the subepidermal zone and the epidermis. (**D**) HE ×100; (**E**) HE ×400. (**F**) A focused view of the dense dermal infiltrate that dissects the collagen. HE ×400. (**G**–**J**,**L**,**M**) Lymph node immunohistochemistry (IHC): tumoral cells are CD45+ (**G**), myeloperoxidase in rare cells (**H**), CD68/PGM1+ (**I**) and CD33+ (**J**). No B or T cell lineage markers found in CD3- (**L**) and CD20- (**M**) at ×20.

Bone marrow evaluation and excisional axillary adenopathy biopsy were performed. The flow cytometry was inconclusive, showing a lack of lineage specificity with CD11c+, CD33+, CD64+ (low), myeloperoxidase (MPO)+ (dubious) and HLA-DR. Bone marrow morphology showed massive infiltration but no clear conclusions due to the extensive immaturity and the aberrant cell morphology (Figure 2C). The patient remained hemodynamically stable with no aggravating symptoms, and he was discharged while awaiting histological results and was closely evaluated as an outpatient. A methylprednisolone pulse regimen (500 mg IV daily for 3 consecutive days) was implemented with no obvious improvement. In the following days, rapid deterioration occurred with asthenia, diffuse skin thickening in the upper trunk, and the appearance of multiple nodules in the neck and upper trunk (Figure 1G–I). Nodules ranged between 1 and 2 cm, were firm in consistency and were non-tender. Following a skin biopsy, the patient was admitted to symptom control. On the 19th day after clinical onset, the bone marrow biopsy was compatible with AML, revealing extensive infiltration by blast cells with a morphology identical to the population that infiltrated the lymph node structure (Figure 2A–F). Histopathology (lymph node and skin) showed the following immunophenotype: MPO (focal), CD45 (low), CD33 (low), CD4 (low), CD68, CD11c, BCL2 and PAX5, all of which, are compatible with myeloid sarcoma (Figure 2G–M). Fluorescence in situ hybridization showed chromosomal translocation of the MLL rearrangement. At the time, the patient was diagnosed with AML with medullary, ganglionary and cutaneous infiltration and presented ECOG 3. When proposed the option of transference to an intensive chemotherapy center, the patient refused. The patient was prescribed hypomethylating agents (5-azacitidine) and Venetoclax. Whilst waiting for Venetoclax approval, on D2 of the 5-Azacitidine cycle, the patient’s health progressively deteriorated with general oedema and marked cutaneous infiltration as well as aggravated skin thickening in the upper and lower trunk and neck. New purple patches appeared on the lower limb (Figure 1J–L) in addition to analytical aggravation; consequently, the patient suffered multiorgan failure and subsequently died.

## 3. Discussion

The spectrum of AML clinical presentation is variable and highly dependent on the location or the organ involved with leukemia [8]. Very rarely, EM AML can involve lung or pleura with only a few cases reported in the literature [9]. In our case, the thoracic CT-Scan showed focal pleural thickening and nodular formation probably related to the underlying disease; nevertheless, a lung biopsy was not performed to confirm this suspicion.

The majority of extramedullary blastic tumors can be classified as pre-B, pre-T or MS using a panel of monoclonal antibodies including myeloid (MPO, lysozyme, CD68 and CD43), together with B and T lineage markers. Staining for MPO was conventionally used to help differentiate lesions from lymphoma. MS is a rare entity, which may occur as the presenting feature before, or concurrent with, the onset of overt Leukemia, resulting in frequent misdiagnosis. The expression of blast cell CD56 (neural cell adhesion molecule) has been associated with both MS and LC [5,10]. The rarity and morphological similarity between blasts and lymphoma cells, especially in the blastic and undifferentiated variants of MS, are considered the main reasons for misdiagnoses. Therefore, correct pathological diagnoses made in good time are essential and requires the pathologist to maintain a high degree of vigilance. Immunohistochemistry shows CD68/PG-M1 as the most commonly expressed marker, with variable expressions of MPO, CD117, CD99, lysozyme, CD34, terminal deoxynucleotidyl transferase, CD56, CD61, CD30, glycophorin A and CD4 [11]. Bone marrow biopsy should be performed in all patients with MS. In our case, the atypical initial presentation with bilateral lymph node enlargement was an added complication to diagnosis. Additionally, the aggression of aberrant cells with complex morphology and the extensive infiltration in bone marrow, lymph node and skin made diagnosis difficult, as the flow cytometry and immunohistochemical stains were also not conclusive, which led to a delayed diagnosis. Certain cytogenetic abnormalities have been associated with a higher incidence of MS [5,12]. The MLL gene mutation has been linked to extramedullary involvement, and prognosis remains poor [13]. In this clinical report, the patient presented a MLL gene mutation as the only cytogenetic irregularity. The treatment paradigm for EM AML follows the same standard treatment for AML in general and depends on the localization of the disease, on whether it is an initial diagnosis or a relapse, on performance status and on the patient’s age [7]. Systemic chemotherapy using AML-like regimens is the first choice of treatment and should be started early, even in non-leukemic diseases. Until recently, AML regimens were restricted to combination-intensive chemotherapies in patients who could tolerate such therapies. Nonetheless, less intensive therapies such as hypomethylating agents have been recently considered in patients with AML who are unsuitable for intensive chemotherapy. The newly approved regimen using a combination of Venetoclax and hypomethylating agents (VEN-HMA) has produced high response rates in patient who are older or unfit with newly diagnosed AML as well as in patients with relapsed/refractory AML [14,15,16]. The response data for VEN-HMA are mainly derived from patients with bone marrow involvement, but the impact of VEN-HMA on the subset of AML with EM remains largely unknown. Recently, the activity of only VEN-HMA in these patients was described as promising and a viable option for EM AML patients who are ineligible for or have failed prior chemotherapy [14]. Treatment options for newly diagnosed EM AML with concurrent bone marrow, lymph node and skin symptoms, together with underlying health issues, has not been described in the literature, to our knowledge. Our patient was treated in a Peripheral Hospital without an Acute Leukemia Unit. The patient refused transference. Given the performance status of the patient at the start of the treatment and the available chemotherapy regimens, the best treatment offered to this patient was the use of VEN-HMA. Unfortunately, the clinical status of the patient was aggravated and he died before starting the treatment.

## 4. Conclusions

EM involvement in AML is a relatively rare but clinically significant occurrence that often causes therapeutic and diagnostic dilemmas for both hematologists and pathologists. A history of AML will point towards this as a diagnosis, but prior knowledge of the condition as well as a high degree of suspicion are essential. Our case demonstrates how challenging the diagnosis of AML may be when presenting with atypical extramedullary disease and highlights how important it is to obtain an early and accurate diagnosis. To the best of our knowledge, there are no case reports in the literature combining EM AML with concurrent bone marrow, lymph node and skin involvement, thus establishing it as a rare entity with poor prognosis. The current therapeutic recommendations are based on extrapolation from medullary AML but further studies targeting EM AML are warranted.

## Figures and Tables

**Table 1:** Relevant laboratory analysis performed at different timepoints (hospital admission, hospital discharge, day hospital, clinical progression and normal values).

Parameter	Hospital Ad. (26 December 2020)	Hospital Dis. (30 December 2020)	Day Hospital (20 January 2021)	Clinical Progression (22 January 2021)	Normal Values
Hb (g/dL)	9.8	9.5	7.8	7.0	13.0–17.5
WBC (uL)	3420	3390	24,270	3430	4.4–11.1
Neutrophils (uL)	300	400	680	190	2.0–7.1
Platelets (uL)	101,000	204,000	127,000	118,000	155–395
C-reactive protein (mg/dL)	3.15	2.86	7.05	8.86	0.0–0.5
LDH (mg/dL)	1144	852	1426	1661	120–246
Creatinine (mg/dL)	0.77	0.93	1.81	1.84	0.7–1.3
Uric Acid (mg/dL)		9.5		11.2	3.5–7.2

## Data Availability

Not applicable.
